# The Effect of Chronic Antipsychotic Drug on Hypothalamic Expression of Neural Nitric Oxide Synthase and Dopamine D2 Receptor in the Male Rat

**DOI:** 10.1371/journal.pone.0033247

**Published:** 2012-04-13

**Authors:** Xiang Rong Zhang, Ying Xin Wang, Zhi Jun Zhang, Lei Li, Gavin P. Reynolds

**Affiliations:** 1 Department of Neuropsychiatry, Affiliated Zhongda Hospital of Southeast University, Nanjing, China; 2 Neuropsychiatric Research Institute, School of Medicine, Southeast University, Nanjing, China; 3 Department of Psychiatry, School of Medicine and Dentistry, Queen’s University Belfast, United Kingdom; 4 Biomedical Research Centre, Sheffield Hallam University, Sheffield, United Kingdom; Baylor College of Medicine, United States of America

## Abstract

Antipsychotic-induced sexual dysfunction is a common and serious clinical side effect. It has been demonstrated that both neuronal nitric oxide (nNOS) and dopamine D2 receptor (DRD2) in the medial preoptic area (MPOA) and the paraventricular nucleus (PVN) of the hypothalamus have important roles in the regulation of sexual behaviour. We investigated the influences of 21 days’ antipsychotic drug administration on expression of nNOS and DRD2 in the rat hypothalamus. Haloperidol (0.5 mg/kg/day i.p.) significantly decreased nNOS integrated optical density in a sub-nucleus of the MPOA, medial preoptic nucleus (MPN), and decreased the nNOS integrated optical density and cell density in another sub-nucleus of the MPOA, anterodorsal preoptic nucleus (ADP). Risperidone (0.25 mg/kg) inhibited the nNOS integrated optical density in the ADP. nNOS mRNA and protein in the MPOA but not the PVN was also significantly decreased by haloperidol. Haloperidol and risperidone increased DRD2 mRNA and protein expression in both the MPOA and the PVN. Quetiapine (20 mg/kg/day i.p.) did not influence the expression of nNOS and DRD2 in either the MPOA or the PVN. These findings indicate that hypothalamic nNOS and DRD2 are affected to different extents by chronic administration of risperidone and haloperidol, but are unaffected by quetiapine. These central effects might play a role in sexual dysfunction induced by certain antipsychotic drugs.

## Introduction

Antipsychotic-induced sexual dysfunction is a common and serious clinical side effect, which is gaining increasing attention within the past decade. Sexual dysfunction has important implications for satisfaction with sexual life and overall quality of life [1], [2]. It is one of the major reasons for treatment noncompliance and inevitably affects overall clinical outcome and treatment success [3], [4], [5].

The mechanism of antipsychotic drug-induced sexual dysfunction is complex and remains unclear. Generally, the sensory information related to sexual behaviour is processed at various brain nuclei, which balance the inhibitory and excitatory influence on spinal sympathetic and parasympathetic centres, and then determine the functional state of the sexual effector organ [6]. Antipsychotics might act both centrally and peripherally to induce sexual dysfunction. We have shown that some antipsychotics change NOS activity and expression in penile tissues [7], as well as demonstrating functional effects on male sexual behaviour in the rat [8], [9]. Research directly investigating the central mechanisms of antipsychotic-induced sexual dysfunction is particularly rare. Understanding of these mechanisms is mostly theoretical, deriving from general knowledge of sex physiology and psychopharmacology, and is generally unverified by basic or clinical investigation.

The medial preoptic area (MPOA) and the paraventricular nucleus (PVN) are two critical brain structures for male sexual behaviour. These nuclei receive direct and indirect input from every sensory modality and send projections to extra-hypothalamic brain areas for the initiation and patterning of copulation [10], [11]. The MPOA and PVN also have mutual connectivity [12], [13]. Neuroanatomical studies further indicated that the MPOA could be divided into several sub-regions, which have different roles in the regulation of sexual function. Lesion studies have inferred that the caudal MPOA could impair copulation more severely than the rostral MPOA while the dorsal MPOA (anterodorsal preoptic nucleus, ADP) may be more important than the medial and other MPOA regions for copulatory behaviour [14], [15].

Previous studies have indicated that dopamine and nitric oxide (NO) might be two of the most important neuromodulators with facilitative effects on sexual function in both the MPOA and the PVN [16]. A direct dopamine D2 receptor antagonist effect has been proposed as the primary underlying mechanism of sexual dysfunction after antipsychotic drug administration [17]. Haloperidol, a dopamine D2 receptor antagonist, has been found to impair sexual behaviour after acute microinjection into the MPOA [18] and the PVN [11]. However, the role of dopamine D2 antagonism has not been tested with other antipsychotic drugs, notably the newer atypical drugs.

The importance of NO in sexual function has been demonstrated by the observation that a NO precursor (L-arginine) facilitates male sexual function, while a NOS inhibitor (L-nitroarginine methyl ester, L-NAME) injected into either the MPOA [10] or the PVN [19], reduces it. An early study suggested that haloperidol could inhibit neuronal nitric oxide synthase (NOS) activity by preventing electron transfer [20], while apomorphine, a mixed D1/D2 agonist, increased NO production in the PVN, which was correlated with penile erection. Acute haloperidol (0.5 mg/kg i.p.) prevented apomorphine’s effect on both NO_2_^−^ concentration and penile erection [21]. Therefore, the NO pathway might also be involved in the development of antipsychotic-induced sexual dysfunction.

The current study investigated the expression of neural NOS (nNOS) and the dopamine D2 receptor in the MPOA and PVN after chronic systemic administration of the typical antipsychotic haloperidol, and the atypical antipsychotics risperidone and quetiapine, in which haloperidol and risperidone, but not quetiapine are associated with a high incidence of sexual dysfunction in both humans [22], [23] and rats [9].

## Methods

### Animals

3-month-old male Sprague-Dawley rats (Shanghai Laboratory Animal Centre, China) were used. After being quarantined for 1 week, animals were housed five or six to a cage with free access to food and water, at an ambient temperature of 18–22°C and 60% humidity with a 12-h light/dark cycle (lights on 7.00AM – 7.00PM). The animals were handled daily. All procedures in animal experiments were approved by the Animal Study Committee of Southeast University, China (ethics approval number 2006ZDLL009.0).

### Drug Administration and Experimental Design

Four groups of 3-month-old male Sprague-Dawley rats (n = 7 in each group) received intraperitoneal injection of haloperidol (0.5 mg/kg), risperidone (0.25 mg/kg), quetiapine (20 mg/kg) or vehicle (saline) once daily for 21 days. The haloperidol dose was chosen to obtain effective receptor occupancy (Wadenberg et al, 2001); does for risperidone and quetiapine were chosen on the basis of their relative clinical potency (Jibson and Tandon, 1998). Risperidone (Sigma, Poole, UK), haloperidol (Seranace liquid, 2 mg/ml) and quetiapine (AstraZeneca, Macclesfield, UK) were dissolved in a few drops of glacial acetic acid, diluted with distilled water and buffered back with NaOH to pH 6.0. The vehicle was saline previously acidified to pH 6.0. The experiment was performed three times for immunocytochemistry, gene expression and protein expression.

### Immunohistochemistry of nNOS

At the end of the drug administration, the rats were sacrificed by deep anaesthesia with 30 mg/kg pentobarbital i.p. followed by transcardial perfusion with 4% paraformaldehyde (PFA) in 0.1 M phosphate buffered saline (PBS). Brains were removed and fixed in 4% PFA for 4 days before being embedded in wax. Three sections (7 µm) from MPOA between Bregma −0.30 and −0.40 mm, determined using the rat brain stereotaxic atlas [24], were mounted onto slides coated with 3-aminopropyltriethoxysilane (APES) (Sigma). PVN sections were taken from Bregma −1.60 to – 1.88 mm. Avidin-Biotin Complex (ABC) method was used to stain for nNOS immunoreactivity. Briefly, following clearing in xylene and rehydration in graded alcohol, sections were incubated in hydrogen peroxide solution to inhibit endogenous peroxidase activity and a microwave treatment protocol was employed to aid antigen retrieval. Sections were then blocked with 10% normal horse serum diluted in 0.01 M PBS. The sections were incubated with a mouse anti-rat polyclonal primary antibody raised against the C-terminal of nNOS protein (1095–1289) (Product NO 610308, BD Transduction Laboratories, USA) at a dilution of 1∶6000 in antibody dilution buffer (10% normal serum in 0.01 M PBS) overnight at 4°C, followed by incubation with biotinylated secondary antibody (anti-mouse IgG) (Vector Laboratories, Burlingame, CA) at a dilution of 1∶200 for 2 h at room temperature. Sections were then incubated for 2 h at room temperature with ABC solution (Vectastain elite kit, Vector Laboratories, Burlingame, CA) and peroxidase was stained by incubation with 3,3′-diaminobenzidine (DAB) solution containing nickel chloride. No immunoreactivity could be detected in control sections, in which the primary antibody was omitted from the staining protocol.

The composite images containing total left and right MPOA and PVN were captured using a RGB JVC solid-state camera connected to an Olympus BH2 microscope at 10-fold objective magnification fitted with a motorized stage. Then two immunohistochemical parameters, the neural positive cell density and integrated optical density (IOD), were analysed using Image-pro plus software (version 4.1, Diagnostic, Inc.) in three horizontal sections (28 µm apart) in each structure. The quantification processes were set blind to section code until the analysis was finished. Two sub-nuclei, anterodorsal preoptic nucleus (ADP) and medial preoptic nucleus (MPN), were investigated in the MPOA, with ADP and MPN defined by a 0.2 × 0.3 mm^2^ and 0.4 × 0.6 mm^2^ rectangle area respectively [25]. The PVN field area was defined by the area within its clear border after DAB staining. Stained neurons were counted using a manual counting option, and then calculated as neuronal density (cells/mm^2^). Integrated optical density (IOD) is the summation of total pixel optical density in the sub-nuclei [26], [27], [28], [29].

### nNOS and DRD2 mRNA Measurement

Brains from each animal were immediately frozen in liquid nitrogen and then allowed to thaw slightly on ice. The MPOA slice was made by cutting between the anterior and posterior edges of the optic chiasm. The tissue containing the MPOA was isolated by cutting laterally to the lateral sulcus and to the depth of the anterior commissure [30]. The PVN slice was made by cutting rostral to the optic chiasm and again 2 mm posterior to the first cut. The tissue containing the PVN was dissected out by cutting 1.5 mm lateral to the median line and undercutting at a depth of 3 mm [31]. The tissues were finally stored at −80°C until analysis.

Total RNA was isolated from the tissue after homogenization using phenol- chloroform TRIzol (Sigma, USA). Genomic DNA contaminants were removed by treatment of the tissue extract with RNase-free DNase (Promega, USA). First-strand cDNA was then synthesized from 2 µg total RNA by incubating with Oligo(dT)15 Primer and M-MLV Reverse Transcriptase according to the manufacturer’s protocol (Promega, USA).

The expression level of nNOS and DRD2 mRNA was examined by a quantitative real-time PCR method using an ABI Prism 7000 Sequence Detector (Applied Biosystems, USA). β-actin was used as the endogenous control. Rat-specific oligonucleotide primers were designed and synthesized by Invitrogen (China) as following: nNOS: FWD 5′-TCA AAG CCA TCC AGC GCA TA-3′, REV 5′-GCG GTT GGT CAC TTC ATA CGT TC-3′; DRD2: FWD 5′-AGA CGA TGA GCC GCA GAA AG -3′, REV 5′-GCA GCC AGC AGA TGA TGA AC-3′; β-actin: FWD 5′-GGA GAT TAC TGC CCT GGC TCC TA-3′, REV 5′-GAC TCA TCG TAC TCC TGC TTG CTG-3′. A final 20 µl reaction was prepared with 10 µl SYBR Green Premix Ex Taq (TaKaRa, Japan), 0.4 µl ROX Reference Dye, 0.8 µl primer pairs, 6.8 µl sterile water and 2 µl cDNA sample. Samples were analysed in triplicate, while a negative control using water as template was included for each primer pair. The PCR reaction was started with an initial enzyme activation step at 95°C for 10 seconds followed by 40 cycles of denaturation at 95°C for 5 seconds and annealing/extension at 62°C for 34 seconds. Data were evaluated by the relative quantification method with efficiency calibrated mathematical model using the control group as a calibrator for comparison of every unknown sample gene expression level [32]. The conversion between Ct and relative gene expression level is n fold induction 2-Ct relative to β-actin.

### Western Blotting Measurement of nNOS and DRD2

The isolated samples were homogenized in lysis buffer (containing 50 mM Tris-HCl, 150 mM NaCl, 0.02 NaN2, 1 mM PMSF, 1 µg/mL Aprotitin and 1% Triton X-100) in the presence of protease inhibitors (5 µL/mL of protease inhibitor cocktail, Sigma). After centrifugation (12,000g, 25 min, 4°C), protein concentration of the supernatant was estimated by the method of Bradford. About 50 µg total protein was then boiled in sample buffer for 5 min and separated by SDS-PAGE using 10% polyacrylamide gels. After electrophoresis, the proteins were transferred to PVDF membranes (Millipore) for 120 min. The membranes were treated with a blocking buffer (5% nonfat dry milk with 0.1% Tween-20) and incubated with primary nNOS and DRD2 antibody respectively (mouse monoclonal antibody against nNOS 1∶500, Transduction Laboratories, Lexington, KY; Rabbit polyclonal antibody against DRD2 1:200, Chemicon International Inc., Temecula, CA) at 4°C overnight. The blots were incubated with a peroxidase-conjugated anti-mouse IgG (Santa Cruz). The reactive bands were detected with an enhanced chemiluminescence method (ECL; Amersham Biosciences). Quantitation of protein bands was carried out by optical densitometry. β-tubulin was used as a control.

### Statistical Analysis

Positive cell density, integrated optical density and mRNA expression were expressed as mean ± s.e.means and were analysed by one-way ANOVA followed by post-hoc Dunnett’s t-test. p<0.05 was considered statistically significant.

## Results

### Immunohistochemistry of nNOS

#### nNOS positive cell density

**Figure 1:**
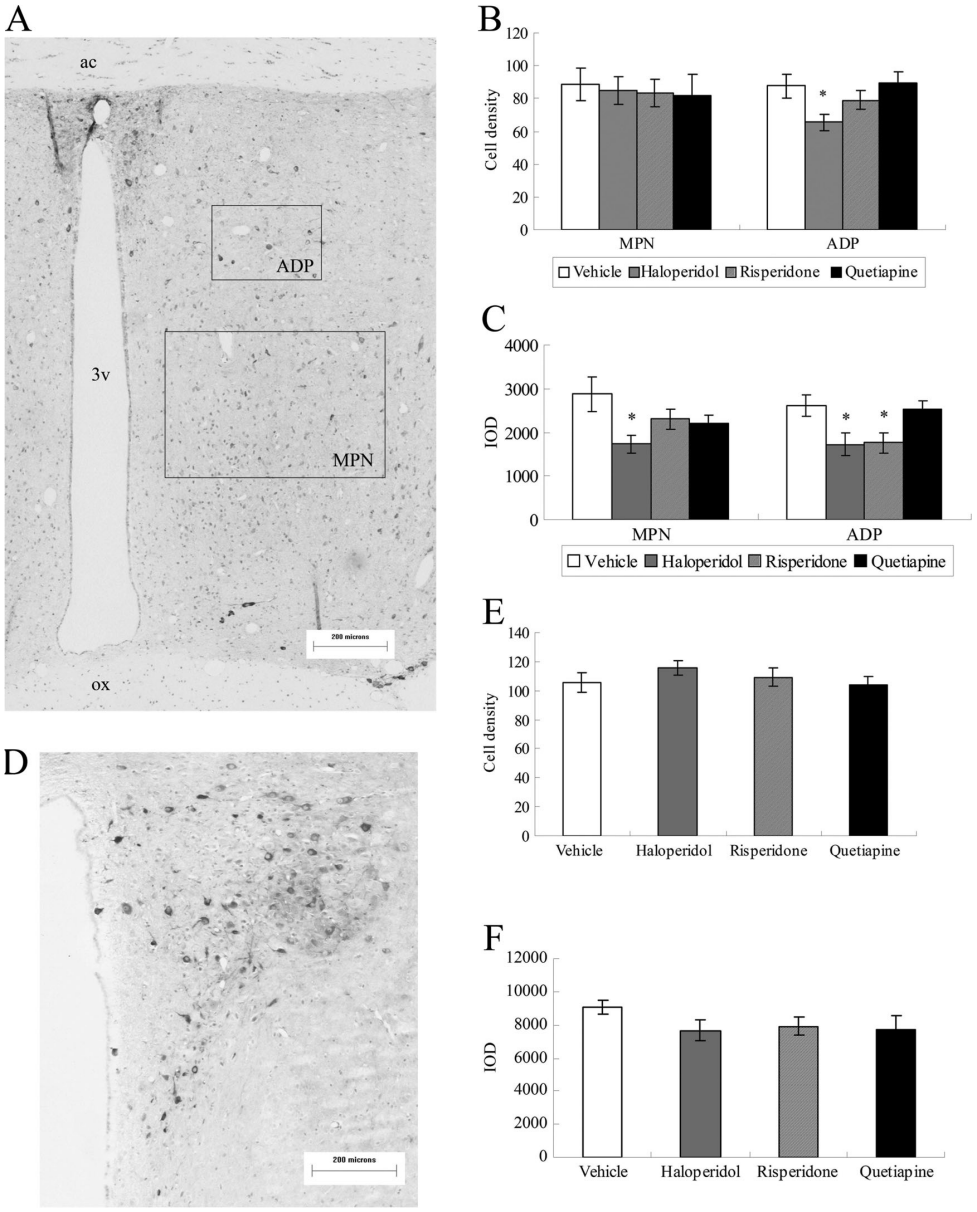
Immunohistochemistry of nNOS in the MPOA and PVN following three weeks of antipsychotic drug administration. nNOS immunostaining in the MPOA (A) and the PVN (D); Quantitative analyses of nNOS positive cell density in the MPOA (B) and the PVN (E), Haloperidol significantly decreased the nNOS positive cell density in the ADP; Quantitative analyses of nNOS IOD in the MPOA (C) and the PVN (F). Haloperidol significantly decreased nNOS IOD in MPN and ADP, and risperidone inhibited the nNOS IOD in the ADP. 3 v: third ventricle; ac: anterior commissure; ADP: anterodorsal preoptic nucleus; ox: optic chiasma; IOD: integrated optical density; MPN: medial preoptic nucleus. Scale bar:200 µm. *p<0.05 vs Vehicle.

The effects of antipsychotic drugs on nNOS positive cell density in the MPOA and PVN are shown in Figure 1. One-way ANOVA showed no significant effect of antipsychotic drug on nNOS positive cell density after three weeks’ administration in the MPN [*F*_(3, 24)_ = 0.081, *p* = 0.970] but revealed a significant between-groups effect on nNOS positive cell density in ADP [*F*_(3,24)_ = 3.743, *p* = 0.024]. Individual comparisons with the vehicle group revealed that nNOS positive cell density in the ADP was significantly reduced by haloperidol (*p* = 0.033), but not by risperidone (*p* = 0.668) or quetiapine (*p* = 0.997). No significant effect of antipsychotic drug on nNOS positive cell density was found in the PVN [*F*_(3, 24)_ = 0.812, *p* = 0.500].

#### Integrated optical density of nNOS staining

The effects of antipsychotic drugs on nNOS integrated optical density (IOD) in the MPOA and PVN are shown in Figure 1. One-way ANOVA revealed a significant between groups effect in the MPN [*F*_(3,24)_ = 3.125, *p* = 0.045]. Individual comparisons with the vehicle group revealed that IOD of nNOS staining was significantly reduced by three weeks’ administration of haloperidol (p = 0.015), but not by risperidone (*p* = 0.212) or quetiapine (*p* = 0.320). A significant effect was also seen in the ADP [*F*_(3, 24)_ = 4.016, *p* = 0.019]. Individual comparisons with vehicle group revealed that IOD of nNOS staining in the ADP was significantly reduced by three weeks administration of haloperidol (*p* = 0.038) and risperidone (*p* = 0.049), but not by quetiapine (*p* = 0.989). No significant effect of antipsychotic drug on IOD was found between groups in the PVN [*F*_(3, 24)_ = 1.089, *p* = 0.373].

### Relative Gene Expression of nNOS and DRD2

#### nNOS

**Figure 2:**
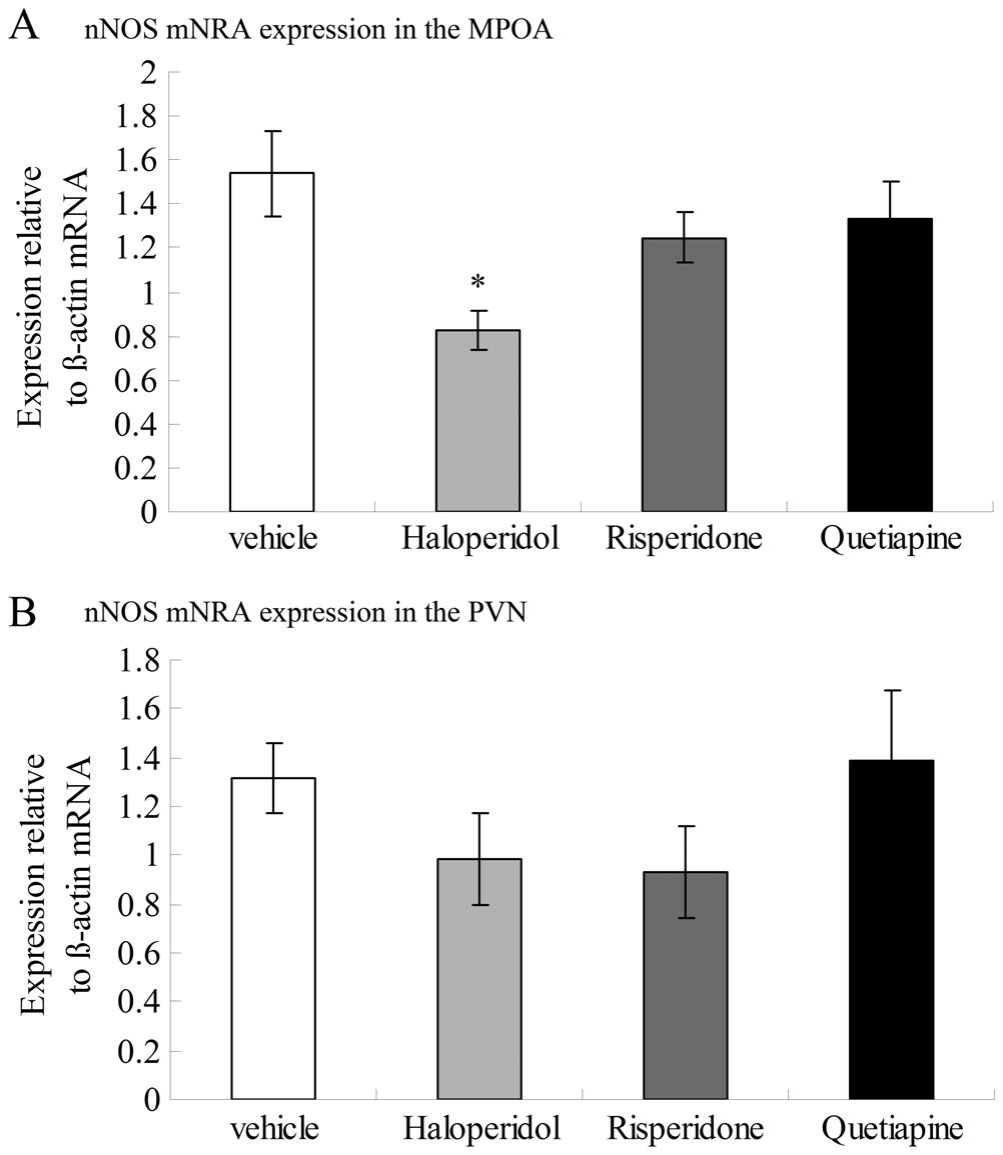
Expression of nNOS mRNA in the MPOA (A) and the PVN (B) following three weeks of antipsychotic drug administration. nNOS mRNA in the MPOA but not the PVN was significantly decreased by haloperidol. Data were relative to β-actin mRNA. *p<0.05 vs Vehicle.

The effects of antipsychotic drugs on nNOS mRNA expression in the MPOA and PVN are shown in Figure 2. One-way ANOVA revealed a significant effect of antipsychotic administration on nNOS mRNA expression [*F*_(3,24)_ = 4.213, *p* = 0.016] in the MPOA. Individual comparisons with the vehicle group revealed that nNOS mRNA expression in the MPOA was significantly reduced by administration of haloperidol (*p* = 0.006) but not by risperidone (*p* = 0.374) and quetiapine (*p* = 0.644). No significant effect of antipsychotic drug on nNOS mRNA expression was found between groups in the PVN [*F*_(3, 24)_ = 1.089, *p* = 0.373].

#### DRD2

**Figure 3:**
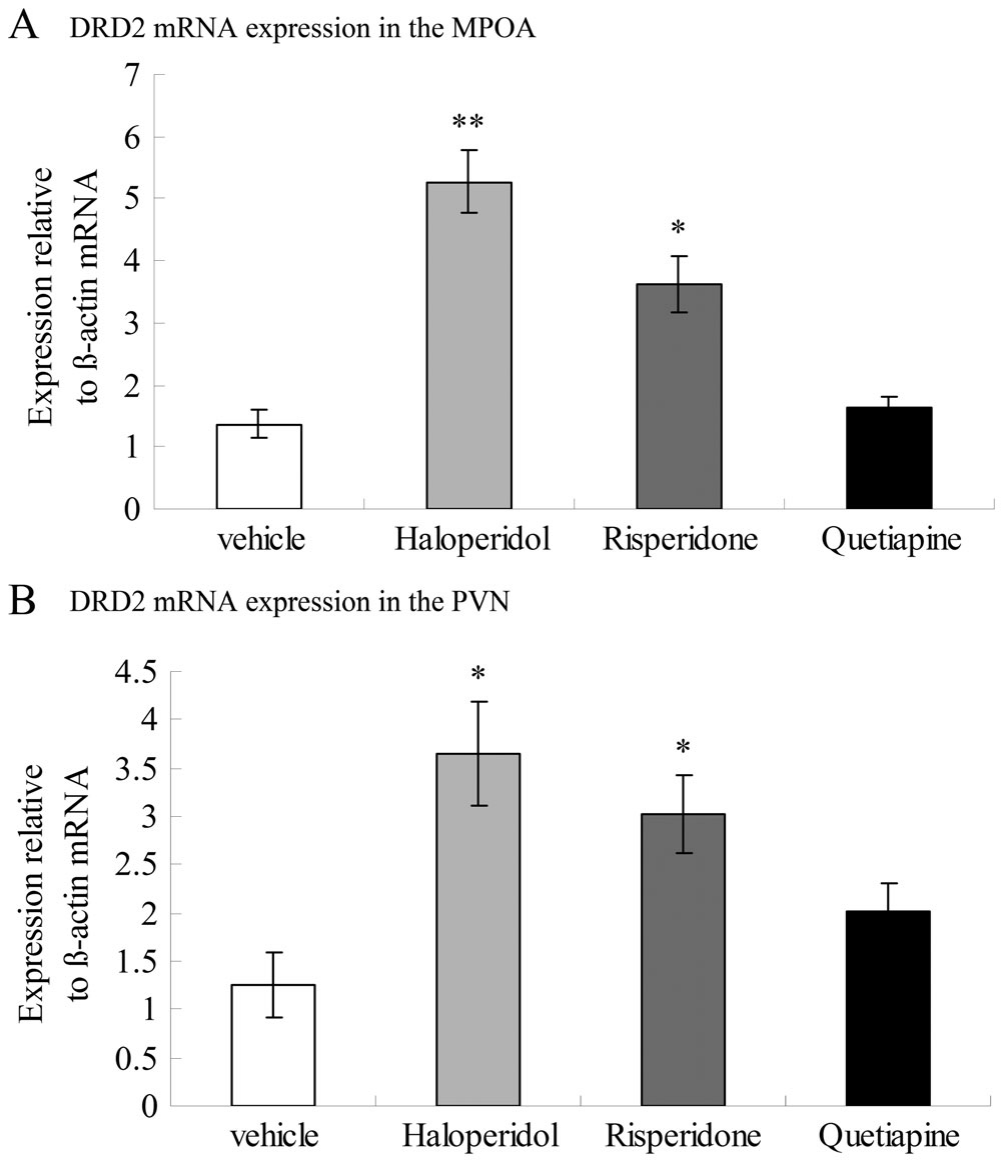
Expression of DRD2 mRNA in the MPOA (A) and the PVN (B) following three weeks of antipsychotic drug administration. Haloperidol and risperidone increased DRD2 mRNA expression in both the MPOA and the PVN. Data were relative to β-actin mRNA. *p<0.05 vs Vehicle.

The effects of antipsychotic drugs on DRD2 mRNA expression in the MPOA and the PVN were shown in Figure 3. One-way ANOVA revealed a significant effect of antipsychotic administration on DRD2 mRNA expression [*F*_(3,24)_ = 23.691, *p*<0.001] in the MPOA. Individual comparisons with vehicle group revealed that DRD2 mRNA expression in the MPOA was significantly increased by three weeks administration of haloperidol (*p*<0.001) and risperidone (*p* = 0.001), but not by quetiapine (*p* = 0.929).

One-way ANOVA also revealed a significant effect of antipsychotic administration on DRD2 mRNA expression [*F*_(3,24)_ = 6.947, *p = *0.002] in the PVN. Individual comparisons with vehicle group revealed that DRD2 mRNA expression in the PVN was significantly increased by administration of haloperidol (*p* = 0.001) and risperidone (*p* = 0.013), but not by quetiapine (*p* = 0.412).

### Protein Expression of nNOS and DRD2

#### nNOS

**Figure 4:**
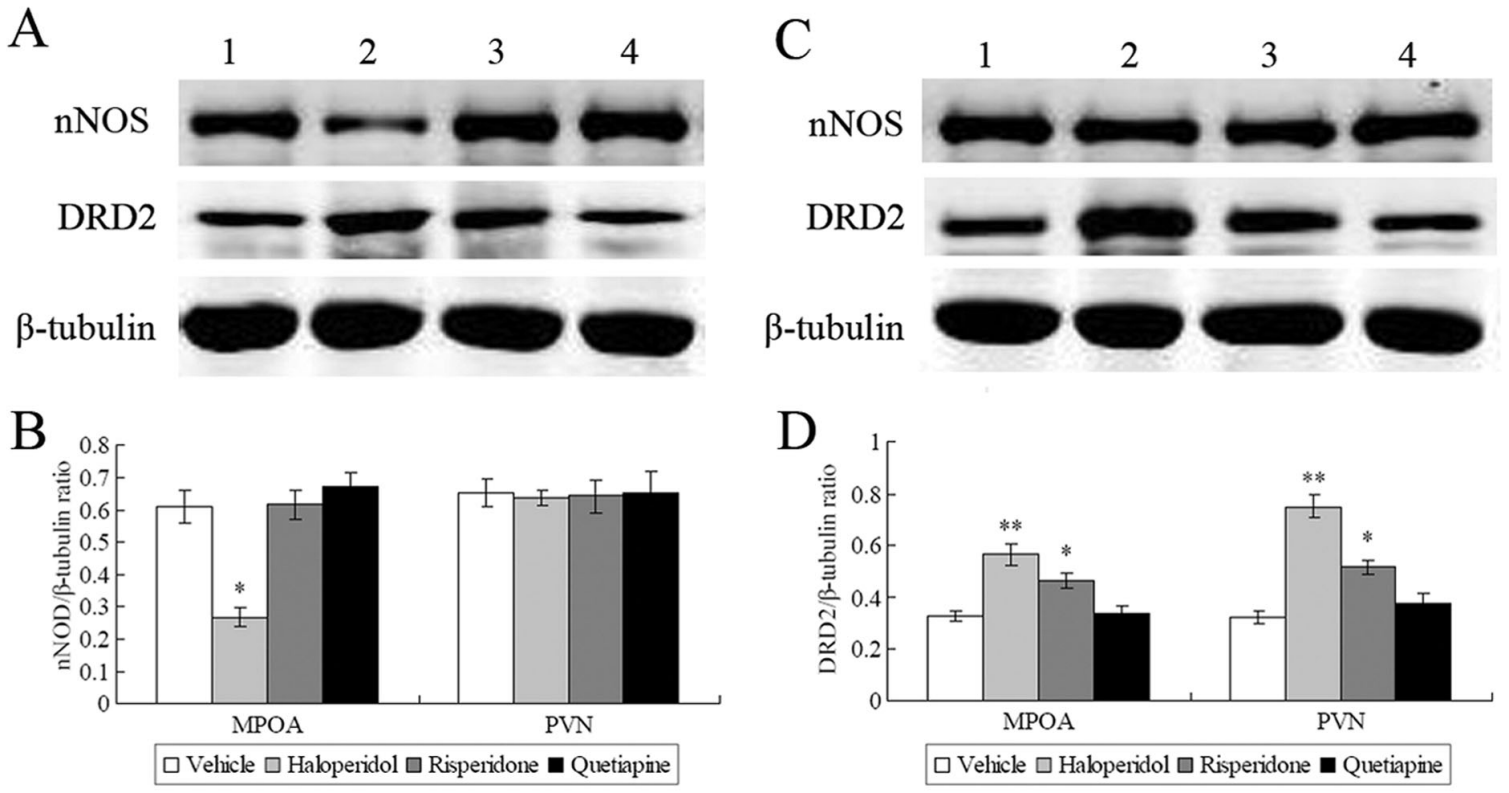
Representative western blot images of nNOS and DRD2 protein expression in the MPOA (A) and the PVN (C) following three weeks of antipsychotic drug administration. The calculated ODs of nNOS (B) and DRD2 (D) protein bands were normalized to each β-tubulin OD and expressed as a ratio. nNOS protien in the MPOA but not the PVN was significantly decreased by haloperidol. Haloperidol and risperidone increased DRD2 protien expression in both the MPOA and the PVN. Lanes: 1 = Vehicle; 2 = haloperidol; 3 = risperdone; 4 = quetiapine. Data are means ± s.e.means and expressed in arbitrary units. *p<0.05 vs Vehicle; **p<0.001 vs Vehicle.

The nNOS monomers display sizes corresponding to approximately 155 kDa, whileβ-tubulin has a size of approximately 55 kDa. One-way ANOVA revealed a significant effect of antipsychotic administration on nNOS expression in the MPOA [*F*_(3,24)_ = 18.115, *p*<0.001] but not the PVN [*F*_(3,24)_ = 0.027, *p* = 0.994]. Individual comparisons with vehicle group revealed that nNOS protein expression in the MPOA was significantly decreased by administration of haloperidol (*p*<0.001) but not risperidone (*p* = 0.999) or quetiapine (*p* = 0.619) (Figure 4).

#### DRD2

The DRD2 immunoreactivity identified a protein band corresponding to approximately 48 kDa. One-way ANOVA revealed a significant effect of antipsychotic administration on DRD2 expression in both the MPOA [*F*_(3,24)_ = 13.976, *p*<0.001] and the PVN [*F*_(3,24)_ = 30.737, *p*<0.001]. Individual comparisons with vehicle group showed that DRD2 protein expression was significantly decreased by administration of haloperidol (MPOA: *p*<0.001; PVN: p = 0.01) and risperidone (MPOA: *p*<0.001; PVN: p = 0.002) but not quetiapine (*p*>0.05) (Figure 4).

## Discussion

The present study evaluated a possible relationship between chronic antipsychotic drug administration and the expression of nNOS in regions of the hypothalamus implicated in sexual function. nNOS catalyzes the synthesis of neuronal NO from L-arginine. Behavioural and neurochemical studies indicate that neuronal NO plays a key role in the central mechanism of sexual function. Originally nNOS was considered a constitutively expressed enzyme but accumulating evidence demonstrates that nNOS expression is highly susceptible to regulation by various physiological or pathological conditions [33]. Previous studies indicated that nNOS expression could also be affected by antipsychotic medication, while the reported effects varied widely with different antipsychotic drugs and different brain regions (as summarised in [7]). Our current study is consistent with those previous findings in that haloperidol and risperidone, but not quetiapine, decreased immunopositive neuron density and IOD of nNOS in the ADP and the MPN to different extents.

Along with the inhibitory effect of haloperidol on nNOS immunoreactivity, chronic treatment with haloperidol decreased levels of nNOS mRNA and protein in the MPOA. These findings indicate that haloperidol might suppress nNOS gene transcription and protein expression, which then reduces the production of neuronal NO in the MPOA and finally lead to sexual dysfunction. The mechanism underlying down-regulation of nNOS synthesis following haloperidol administration remains unclear. nNOS is regulated by Ca^2+^ influx through L-type voltage-sensitive Ca^2+^ channels [33], while haloperidol has been demonstrated to inhibit the L-type Ca^2+^ channel at micromolar concentrations [34]. Wu et al., (2000) reported that haloperidol but not risperidone (both 10 uM) could significantly reduce the amplitude of L-type Ca^2+^ current in vitro [35]. Collectively, these studies provide a possible mechanism underlying the effect of haloperidol on nNOS expression. However, these inhibitory effects occurred at relatively high concentrations, and extrapolation to the in vivo situation should be undertaken with caution.

Risperidone also showed an effect on nNOS expression in the MPOA, indicated by a suppression of nNOS IOD in the ADP, although nNOS-immunopositive neuron density was not significantly changed. Risperidone did not significantly influence nNOS mRNA and protein levels in the MPOA; however differences restricted to individual sub-nuclei may not be detectable in this analysis as they may not be robust enough to influence the result in the whole nucleus.

In the hypothalamus, dopamine D2 receptor density is rich in both the MPOA and the PVN [36], [37]. Sexual function was noticeably inhibited after infusions of haloperidol to the MPOA or the PVN [11], [18]. D2 receptor expression in the brain is increased after chronic treatment with antipsychotic drugs [38], [39], while continuous infusion of the dopamine D2 agonist quinpirole can cause a significant down-regulation of striatal D2 dopamine receptors [40]. The alterations of D2 receptor expression also parallel dopamine-mediated behaviours, such as stereotypy and locomotor behaviour [40]. Although the increase in D2 receptors is not always accompanied by an increase in D2 mRNA [41], higher doses and more prolonged exposure to antipsychotic drugs do elevate D2 mRNA [42], [43]. We find that haloperidol and risperidone increased the DRD2 mRNA and protein expression in the MPOA and the PVN. This is the first study providing direct evidence for dopamine D2 receptor alteration induced by antipsychotic drugs in those two neural sites. Our study also provides strong support for the hypothalamic dopamine D2 receptor antagonism hypothesis of antipsychotic drug-induced sexual dysfunction. This hypothesis is also supported by our previous observation of an association of D2 receptor polymorphisms with sexual dysfunction in male subjects receiving antipsychotic treatment [44].

While the current study is limited by using only single dose regimes, these were chosen to be equivalent to clinically-effective doses on the basis of inducing substantial and effective dopamine D2 receptor occupancy. Previous studies from this laboratory have investigated the effects of a range of doses of these three drugs in rat sexual behavior; the doses chosen here were at (haloperidol) or below (risperidone and quetiapine) the threshold dose that impaired aspects of male sexual function [9].

As reviewed by Hull and Dominguez [10], chemosensory information activates the MPOA neurons, resulting in Ca^2+^ influx and NOS activation. The resultant NO production may increase dopamine neurotransmission to facilitate sexual function via D2-like receptors. The incerto-hypothalamic dopaminergic neuron terminals from the MPOA release dopamine in the PVN and then activate NOS production in the oxytocin-containing neurons of the PVN, which in turn facilitates penile erection [11], [19]. According to our current findings, antipsychotic drugs might impair sexual function through the following various mechanisms: 1) down-regulation of nNOS expression, thereby decreasing production of NO and consequently synaptic dopamine in the MPOA and/or 2) blocking the postsynaptic D2 receptor directly in the PVN and consequently inhibiting sexual behaviour. These central effects might be involved in sexual dysfunction induced by certain antipsychotic drugs. These differences of sexual dysfunction induced by various antipsychotic drugs observed in clinical medication are likely because of the different pharmacological properties of these antipsychotics.

## References

[1] Mallis D, Moisidis K, Kirana PS, Papaharitou S, Simos G (2006). Moderate and severe erectile dysfunction equally affects life satisfaction.. J Sex Med.

[2] Olfson M, Uttaro T, Carson WH, Tafesse E (2005). Male sexual dysfunction and quality of life in schizophrenia.. J Clin Psychiatry.

[3] Lambert M, Conus P, Eide P, Mass R, Karow A (2004). Impact of present and past antipsychotic side effects on attitude toward typical antipsychotic treatment and adherence.. Eur Psychiatry.

[4] Perkins DO (2002). Predictors of noncompliance in patients with schizophrenia.. J Clin Psychiatry.

[5] Rosenberg KP, Bleiberg KL, Koscis J, Gross C (2003). A survey of sexual side effects among severely mentally ill patients taking psychotropic medications: impact on compliance.J Sex Marital Ther.

[6] Peeters M, Giuliano F (2008). Central neurophysiology and dopaminergic control of ejaculation.. Neurosci Biobehav Rev.

[7] Zhang XR, Zhang ZJ, Jenkins TA, Cheng WR, Reynolds GP (2010). The effect of chronic antipsychotic drug administration on nitric oxide synthase activity and gene expression in rat penile tissues.. Eur Neuropsychopharmacol.

[8] Zhang X, Zhang Z, Cheng W, Mou X, Reynolds GP (2007). The effect of chronic antipsychotic treatment on sexual behaviour, hormones and organ size in the male rat.. J Psychopharmacol.

[9] Zhang XR, Zhang ZJ, Jenkins TA, Cheng WR, Reynolds GP (2011). The Dose-Dependent Effect of Chronic Administration of Haloperidol, Risperidone, and Quetiapine on Sexual Behavior in the Male Rat.. J Sex Med.

[10] Hull EM, Dominguez JM (2006). Getting his act together: roles of glutamate, nitric oxide, and dopamine in the medial preoptic area.. Brain Res.

[11] Melis MR, Melis T, Cocco C, Succu S, Sanna F (2007). Oxytocin injected into the ventral tegmental area induces penile erection and increases extracellular dopamine in the nucleus accumbens and paraventricular nucleus of the hypothalamus of male rats.. Eur J Neurosci.

[12] Simerly RB, Swanson LW (1988). Projections of the medial preoptic nucleus: a Phaseolus vulgaris leucoagglutinin anterograde tract-tracing study in the rat.. J Comp Neuro.

[13] Simerly RB, Swanson LW (1986). The organization of neural inputs to the medial preoptic nucleus of the rat.. J Comp Neurol.

[14] Balthazart J, Ball GF (2007). Topography in the preoptic region: differential regulation of appetitive and consummatory male sexual behaviors.. Front Neuroendocrinol.

[15] Arendash GW, Gorski RA (1983). Effects of discrete lesions of the sexually dimorphic nucleus of the preoptic area or other medial preoptic regions on the sexual behavior of male rats.. Brain Res Bull.

[16] Argiolas A, Melis MR (2003). The neurophysiology of the sexual cycle.. J Endocrinol Invest,.

[17] Cutler AJ (2003). Sexual dysfunction and antipsychotic treatment.. Psychoneuroendocrinology.

[18] Pfaus JG, Phillips AG (1991). Role of dopamine in anticipatory and consummatory aspects of sexual behavior in the male rat.. Behav Neurosci.

[19] Argiolas A, Melis MR (2005). Central control of penile erection: role of the paraventricular nucleus of the hypothalamus.. Prog Neurobiol.

[20] Iwahashi K, Yoneyama H, Ohnishi T, Nakamura K, Miyatake R (1996). Haloperidol inhibits neuronal nitric oxide synthase activity by preventing electron transfer.. Neuropsychobiology.

[21] Melis MR, Succu S, Argiolas A (1996). Dopamine agonists increase nitric oxide production in the paraventricular nucleus of the hypothalamus: correlation with penile erection and yawning.. Eur J Neurosci.

[22] Dossenbach M, Erol A, el Mahfoud Kessaci M, Shaheen MO, Sunbol MM, et al (2004). Effectiveness of antipsychotic treatments for schizophrenia: interim 6-month analysis from a prospective observational study (IC-SOHO) comparing olanzapine, quetiapine, risperidone, and haloperidol.. J Clin Psychiatry.

[23] Knegtering R, Castelein S, Bous H, der Van L, Bruggeman R (2004). A randomized open-label study of the impact of quetiapine versus risperidone on sexual functioning.. J Clin Psychopharmacol.

[24] Paxinos G, Watson C (1998). The Rat Brain in Stereotaxic Coordinates. fourth ed [M].. San Diego, USA: Academic Press,.

[25] Sato S, Braham CS, Putnam SK, Hull EM (2005). Neuronal nitric oxide synthase and gonadal steroid interaction in the MPOA of male rats: co-localization and testosterone-induced restoration of copulation and nNOS-immunoreactivity.. Brain Res.

[26] Wood RI, Newman SW (1999). Androgen receptor immunoreactivity in the male and female Syrian hamster brain.. J Neurobiol.

[27] Wang-Tilz Y, Tilz C, Wang B, Tilz GP, Stefan H (2006). Influence of lamotrigine and topiramate on MDR1 expression in difficult-to-treat temporal lobe epilepsy.. Epilepsia.

[28] Xi ZQ, Wang XF, He RQ, Li MW, Liu XZ (2007). Extracellular signal-regulated protein kinase in human intractable epilepsy.. Eur J Neurol.

[29] Xavier LL, Viola GG, Ferraz AC, Da CC, Deonizio JM (2005). A simple and fast densitometric method for the analysis of tyrosine hydroxylase immunoreactivity in the substantia nigra pars compacta and in the ventral tegmental area.. Brain Res Brain Res Protoc.

[30] Pu S, Kalra PS, Kalra SP (1998). Ovarian steroid-independent diurnal rhythm in cyclic GMP/nitric oxide efflux in the medial preoptic area: possible role in preovulatory and ovarian steroid-induced LH surge.. J Neuroendocrinol.

[31] Jensen D, Zhang Z, Flynn FW (2008). Trafficking of tachykinin neurokinin 3 receptor to nuclei of neurons in the paraventricular nucleus of the hypothalamus following osmotic challenge.. Neuroscience.

[32] Pfaffl MW (2001). A new mathematical model for relative quantification in real-time RT-PCR.. Nucleic Acids Res.

[33] Sasaki M, Gonzalez-Zulueta M, Huang H, Herring WJ, Ahn S (2000). Dynamic regulation of neuronal NO synthase transcription by calcium influx through a CREB family transcription factor-dependent mechanism.. Proc Natl Acad Sci USA.

[34] Fletcher EJ, Church J, MacDonald JF (1994). Haloperidol blocks voltage-activated Ca2+ channels in hippocampal neurones.. Eur J Pharmacol.

[35] Wu SN, Jan CR, Li HF, Chiang HT (2000). Characterization of inhibition by risperidone of the inwardly rectifying K(+) current in pituitary GH(3) cells.. Neuropsychopharmacology.

[36] Gurevich EV, Joyce JN (1999). Distribution of dopamine D3 receptor expressing neurons in the human forebrain: comparison with D2 receptor expressing neurons.. Neuropsychopharmacology.

[37] Meador-Woodruff JH, Mansour A, Healy DJ, Kuehn R, Zhou QY (1991). Comparison of the distributions of D1 and D2 dopamine receptor mRNAs in rat brain.. Neuropsychopharmacology.

[38] Jenner P, Kerwin R, Rupniak NM, Murugaiah K, Hall MD (1983). Long-term adaptive changes in striatal dopamine function in response to chronic neuroleptic intake in rats.. J Neural Transm Suppl,.

[39] Rupniak MN, Jenner P, Marsden CD (1983). The effect of chronic neuroleptic administration on cerebral dopamine receptor function.. Life Sci,.

[40] Chen JF, Aloyo VJ, Weiss B (1993). Continuous treatment with the D2 dopamine receptor agonist quinpirole decreases D2 dopamine receptors, D2 dopamine receptor messenger RNA and proenkephalin messenger RNA, and increases mu opioid receptors in mouse striatum.. Neuroscience.

[41] Fox CA, Mansour A, Watson SJ (1994). The effects of haloperidol on dopamine receptor gene expression.. Exp Neurol.

[42] D’Souza, U, McGuffin, P, Buckland PR (1997). Antipsychotic regulation of dopamine D1, D2 and D3 receptor mRNA.. Neuropharmacology.

[43] Joyce JN (2001). D2 but not D3 receptors are elevated after 9 or 11 months chronic haloperidol treatment: influence of withdrawal period.. Synapse.

[44] Zhang XR, Zhang ZJ, Zhu RX, Yuan YG, Jenkins TA (2011). Sexual dysfunction in male schizophrenia: influence of antipsychotic drugs, prolactin and polymorphisms of the dopamine D2 receptor genes.. Pharmacogenomics.

